# Purification and Characterization of Asparaginase from *Phaseolus vulgaris* Seeds

**DOI:** 10.1155/2015/309214

**Published:** 2015-08-27

**Authors:** Saleh A. Mohamed, Mohamed F. Elshal, Taha A. Kumosani, Alia M. Aldahlawi

**Affiliations:** ^1^Biochemistry Department, Faculty of Science, King Abdulaziz University, Jeddah, Saudi Arabia; ^2^Biology Department, Faculty of Science, King Abdulaziz University, Jeddah, Saudi Arabia

## Abstract

L-asparaginase from bacteria has been used in treatment of acute lymphoblastic leukemia. The aim of this study was to purify and characterize L-asparaginase from *Phaseolus vulgaris* seeds instead of microbial sources. L-asparaginase was purified to apparent homogeneity. The enzyme has molecular mass of 79 kDa. The purified asparaginase had very low activity toward a number of asparagine and glutamine analogues. L-asparaginase was free from glutaminase activity. Kinetic parameters, Km and *V*max of purified enzyme, were found to be 6.72 mM and 0.16 *μ*M, respectively. The enzyme had optimum pH at 8.0. The enzyme showed high stability at alkaline pH (pH 7.5–9.0) when incubated for up to 24 h. L-asparaginase had the same temperature optimum and thermal stability at 37°C. K^+^ was able to greatly enhance the activity of asparaginase by 150% compared with other metals tested. In conclusion, L-asparaginase showed no glutaminase activity and good stability over a wide range of physiological conditions, and thus it could be used as a potential candidate for treatment of acute lymphoblastic leukemia.

## 1. Introduction

L-asparaginase (L-asparagine amidohydrolase E.C.3.5.1.1) is used in treatment of acute lymphoblastic leukemia and non-Hodgkin's lymphoma [1–3]. The use of L-asparaginase in anticancer therapy is based on its ability to cleave L-asparagine, an amino acid essential for lymphoblasts' growth, to ammonia and L-aspartic acid in serum and cerebrospinal fluid, since lymphoblasts are unable to produce endogenous L-asparagine which leads to death of these cells [4]. Most of the cancer cells are dependent on an exogenous source of this amino acid for survival. However, normal cells are able to synthesize L-asparagine and thus are less affected by its rapid depletion due to treatment with this enzyme. L-asparaginase can also be used to reduce the formation of acrylamide in fried and oven-cooked foods especially in potato chips [5, 6]. The formation of acrylamide was attributed to the reaction of free asparagine and reducing sugars [7]. The depletion of asparagine by asparaginase prevented acrylamide formation [7].

L-asparaginase is widely distributed among plants, animals, and microorganisms [8]. In plant, this enzyme was first detected in the developing seeds of* Lupinus albus* [9]. Asparaginase has also been purified from the testa of maturing seeds of* L. polyphyllus* [10]. Two forms of the enzyme have been identified. A K^+^-independent form is found in* L. arboreus* [11, 12] and* L. polyphyllus* [13] and a K^+^-dependent form is found in* Pisum sativum* and several other legume species, including other* Lupinus* species [14]. Work with antibodies to the K^+^-independent form from* L. polyphyllus* revealed no cross-reaction with either pea asparaginase or a number of varieties of* Lupinus* containing the K^+^-dependent enzyme, suggesting that the two forms of asparaginase are immunologically distinct [15].

Very little information has been reported about using of plant asparaginase in treatment of acute lymphoblastic leukemia [16]. Therefore, the main goal of this study was to purify and characterize L-asparaginase from* Phaseolus vulgaris* seeds free from L-glutaminase instead of L-asparaginase from microbial sources which is used as anticancer and caused side effects due to its immunological responses.

## 2. Material and Methods

### 2.1. Plant Materials

Mature seeds from* Phaseolus vulgaris* cv. Giza 6 were obtained from the Agricultural Research Centre, Cairo, Egypt.

### 2.2. Purification of Asparaginase

#### 2.2.1. Crude Extract

The crude extract of asparaginase was prepared by homogenization of 50 g seeds from* Phaseolus vulgaris* cv. Giza 6 in 20 mM Tris-HCl buffer, pH 8.0 containing 10% glycerol, 50 mM KCl, 12.5 mM *β*-mercaptoethanol, and 1 mM PMSF. The homogenate was centrifuged at 10,000 ×g and the supernatant was designated as crude extract. The crude extract was concentrated by dialysis against solid sucrose.

#### 2.2.2. DEAE-Sepharose Column

Concentrated crude extract was applied onto a DEAE-Sepharose column (15 × 1.6 cm i.d.) which was previously equilibrated with 20 mM Tris-HCl buffer, pH 8.0. The enzyme was eluted by different concentrations of KCl prepared in the same buffer at a flow rate of 60 mL/h and 3 mL fractions were collected. Three peaks of protein were eluted with asparaginase activity according to the elution order (asparaginases I, II, and III).

#### 2.2.3. Sephacryl S-200

Asparaginase I with highest activity was applied onto a Sephacryl S-200 column (90 × 0.6 cm i.d.) which was previously equilibrated with the same buffer at a flow rate of 30 mL/h and 3 mL fractions were collected.

### 2.3. Asparaginase Assay

The activity of L-asparaginase was measured by modified method of Wriston [17]. The L-asparaginase catalyzes L-asparagine to L-aspartic acid and ammonia and the latter react with Nessler's reagent to produce an orange colored product. The enzyme assay mixture consisted of 900 *μ*L of freshly prepared L-asparagine (20 mM) in 50 mM Tris-HCl buffer (pH 8.0), 50 mM KCl, and 100 *μ*L of crude extract of the enzyme. The reaction mixture was incubated at 37°C for 30 min and the reaction was stopped by adding 100 *μ*L of 15% trichloroacetic acid. The reaction mixture was centrifuged at 10,000 ×g for 5 min at 4°C to remove the precipitates. The ammonia released in the supernatant was determined using colorimetric technique by adding 100 *μ*L Nessler's reagent into the sample containing 100 *μ*L supernatant and 800 *μ*L distilled water. The contents in the sample were vortexed and incubated at room temperature for 10 min and OD was measured at 425 nm. The ammonia produced in the reaction was determined based on the standard curve obtained with ammonium sulfate. One unit of L-asparaginase activity is defined as the amount of the enzyme that liberates 1 *μ*mol of ammonia per min at 37°C.

### 2.4. Protein Determination

Protein was quantified by the method of Bradford [18] with bovine serum albumin as standard.

### 2.5. Molecular Weight Determination

The native molecular weight was determined by Sephacryl S-200. The column was calibrated with cytochrome C (12,400), carbonic anhydrase (29,000), bovine serum albumin (67,000), alcohol dehydrogenase (150,000), and *β*-amylase (200,000). Dextran blue (2,000,000) was used to determine the void volume (Vo). Subunit molecular weight was estimated by SDS-polyacrylamide gel electrophoresis [19]. SDS-denatured phosphorylase b (94,000), bovine serum albumin (67,000), ovalbumin (43,000), carbonic anhydrase (30,000), soybean trypsin inhibitor (20,000), and *α*-lactalbumin (14,200) were used for the calibration curve.

### 2.6. Characterization of Asparaginase

#### 2.6.1. Substrate Specificity

Asparaginase activity was determined with some analogs of L-asparagine. The relative activity was expressed as the percentage ratio of the enzyme activity determined against different structure analogs of L-asparagine to enzyme activity with L-asparagine.

#### 2.6.2. Kinetic Parameters

The values of Michaelis constants (Km) and maximum velocity (*V*max) were determined using L-asparagine as substrate in the range of 2–20 mM. Kinetic parameters were determined from Lineweaver-Burk plot.

#### 2.6.3. Effect of pH

The optimum pH for the asparaginase activity was determined by assaying the activity at different pH values. The pH stability was tested by incubation of the enzyme at pH of 5.0–9.0 for 24 h at 4°C in the absence of substrate and residual activity was determined under the standard assay conditions.

#### 2.6.4. Effect of Temperature

The optimum temperature for asparaginase activity was determined by assaying the enzyme at different temperatures. Heat stability was measured by incubating the enzyme alone at different temperatures for 1 h. After heat treatment, the enzyme solution was cooled and the residual activity was assayed after adding the substrates.

#### 2.6.5. Metal Ion Effect

The effects of various metal ions on enzyme activity were determined by preincubating the enzyme alone with 10 mM metal ions for 15 min prior to adding the substrate. The activity which was assayed in the absence of metal ions was taken as 100%.

## 3. Results and Discussion

**Figure 1:**
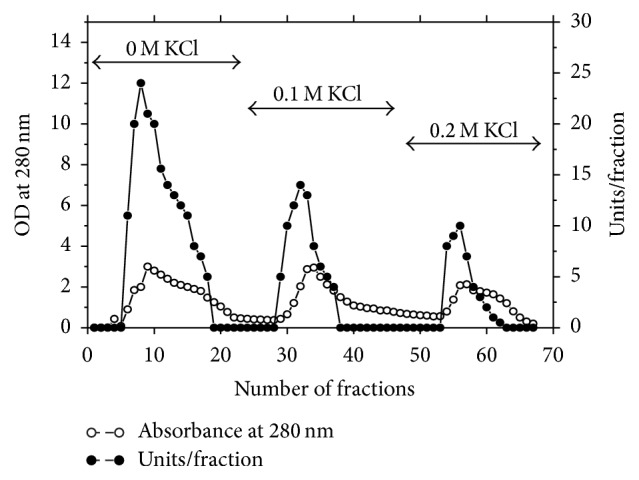
A typical elution profile for the chromatography of asparaginase from* P. vulgaris* on DEAE-Sepharose column (15 × 1.6 cm i.d.) previously equilibrated with 20 mM Tris-HCl buffer, pH 8.0 at a flow rate of 60 mL/h and 3 mL fractions.

**Figure 2:**
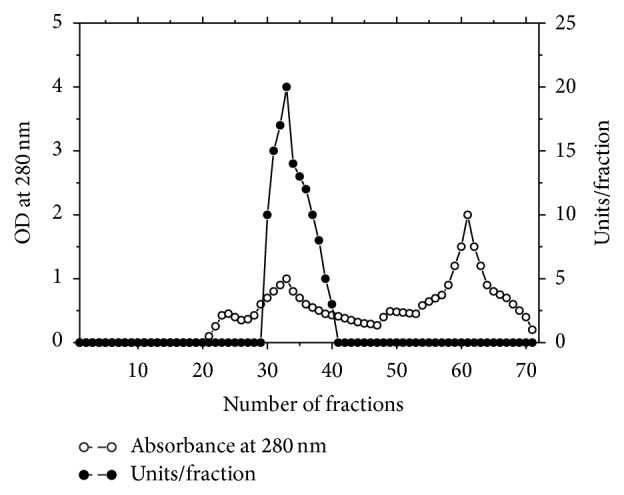
Gel filtration of asparaginase I from DEAE-Sepharose fraction on Sephacryl S-200 column (90 × 1.6 cm i.d.). The column was equilibrated with 20 mM Tris-HCl buffer, pH 8.0 at a flow rate of 30 mL/h and 3 mL fractions.

**Figure 3:**
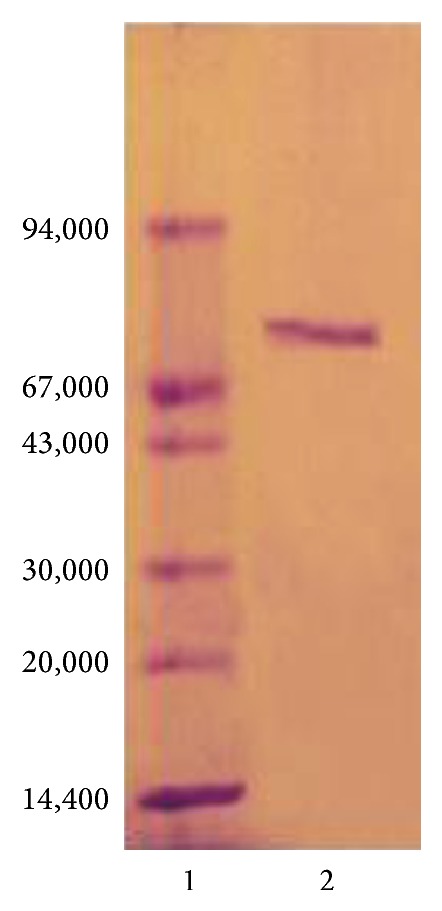
SDS-PAGE for homogeneity and molecular weight determination of asparaginase I from* Phaseolus vulgaris*. (1) Protein markers; (2) Sephacryl S-200 asparaginase I.

The results of purification steps of asparaginase from* P. vulgaris* are summarized in Table 1. The elution profile of the chromatography on DEAE-Sepharose column (Figure 1) showed three peaks of proteins with asparaginase activity. Peak one with the highest asparaginase activity was applied onto a Sephacryl S-200 column (Figure 2). L-asparaginase I was purified 21.7-fold with a specific activity 846 units/mg protein. The asparaginase I was proved to be pure after Sephacryl S-200 column as assessed by SDS-PAGE (Figure 3). The molecular weight of asparaginase I by Sephacryl S-200 and SDS-PAGE procedures yielded a value of 79 kDa as monomer subunit. This finding is in agreement with molecular weights for asparaginases from* Vigna unguiculata* (70 kDa) [20] and* Lupinus polyphyllus* (75 kDa) [15]. A medium molecular weight of 58 kDa was detected for asparaginase from pea leaves [21]. For bacterial asparaginases, the molecular weight ranged from 140 to 160 kDa with tetramer subunits [22–25]. A very low molecular weight of 11.2 kDa was detected for* Streptobacillus* sp. KK2S4 asparaginase [26].

The substrate specificity of asparaginase I has been examined using a number of asparagine and glutamine analogues (Table 2). The activity with the L-asparagine was regarded as 100% activity. DL-asparagine exhibited 30% of enzyme activity, where DL-asparagine is composed of a 1 : 1 racemic mixture. D-asparagine, L-aspartic acid, and L-glutamic acid analogues had very low activity toward asparaginase I. No activity was detected in the presence of L-glutamine. Therefore, the asparaginase I from* P. vulgaris* is free from glutaminase. The contamination of asparaginase with glutaminase activity caused side effects during the course of anticancer therapy [27, 28]. The* L. arboreus* asparaginase hydrolyzed only L-asparagine and DL-aspartyl hydroxamate [12]. The* V. unguiculata* asparaginase was specific for L-asparagine, did not hydrolyze D-asparagine, and was not specific for L-glutamine [20].

**Figure 4:**
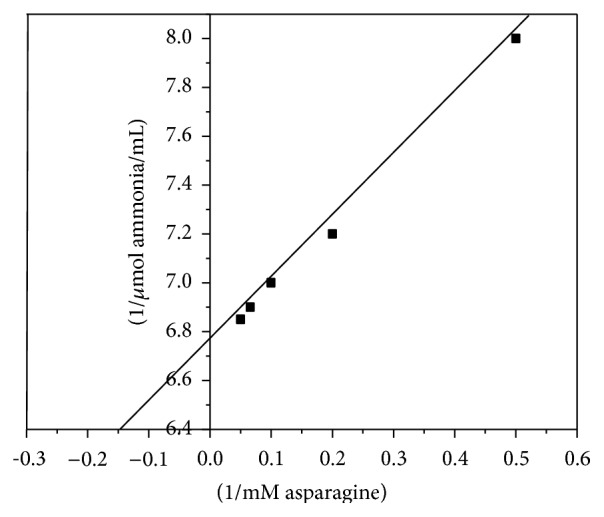
Lineweaver-Burk plot relating of asparaginase I from* P. vulgaris* velocities to different L-asparagine concentrations.

Kinetic parameters, Km and* V*max of purified enzyme, were found to be 6.72 mM asparagine and 0.16 *μ*M ammonia/mL, respectively (Figure 4). Similar Km values of 6.6 and 7.0 mM were determined for asparaginases from* L. arboreus* and* L. angustifolius*, respectively [12]. Asparaginase from* Lupinus* seeds has high Km for asparagine (12.2 mM) [13]. Low Km (1.2 mM) was determined for asparaginase from* V. unguiculata* [20]. For bacteria, Km values for L-asparaginase from* Escherichia coli* and* Erwinia carotovora* were 3.5 and 7.14 mM, respectively [29, 30].

**Figure 5:**
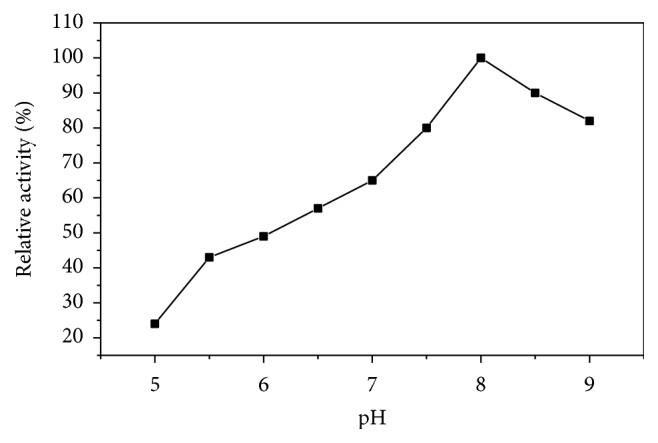
pH optimum of asparaginase I from* P. vulgaris*. The enzyme activity was measured at various pH's using the standard assay method as previously described.

**Figure 6:**
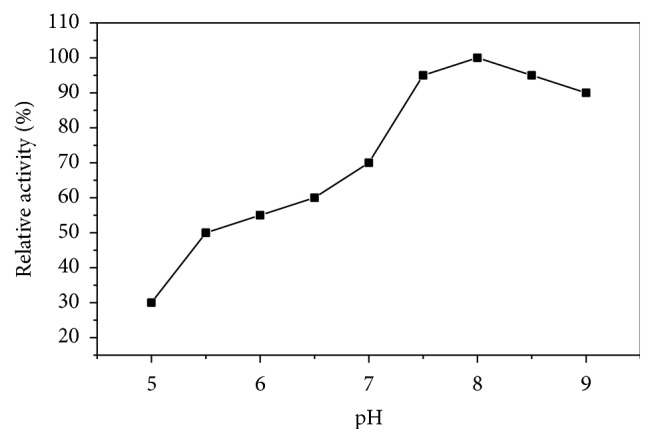
pH stability of asparaginase I from* P. vulgaris* at different pH after incubation for 24 h at 4°C.

Asparaginase I exhibited pH optimum at 8.0 (Figure 5). Between pH 6.0 and 9.0, more than 50% of its activity was retained. Although maximum activity at physiological pH is one of the prerequisites of L-asparaginase for antitumor activity, the purified enzyme would be useful because 80% of the enzyme activity was retained at pH 7.5. The enzyme showed stability at alkaline pH (pH 7.5–9.0) as it retained 90% of its original activity when incubated up to 24 h (Figure 6). However, the pH optimum of L-asparaginases from several plants ranged from 8.0 to 8.5 [12, 20, 31]. Most of L-asparaginases from bacteria showed alkaline pH optima (8.0–10) [26, 30, 32, 33].

**Figure 7:**
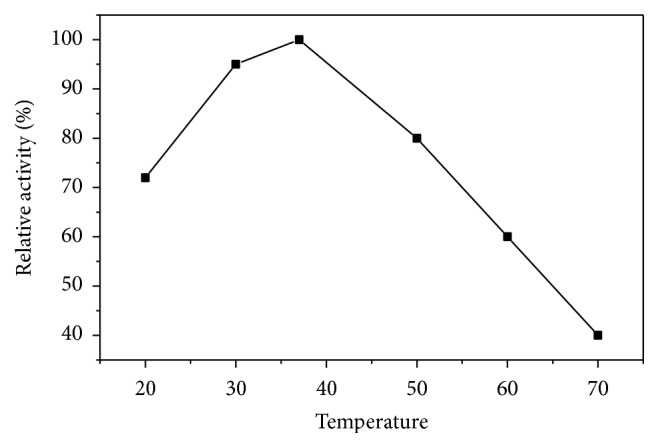
Temperature optimum of asparaginase I from* P. vulgaris*. The enzyme activity was measured at various temperatures using the standard assay method as previously described.

**Figure 8:**
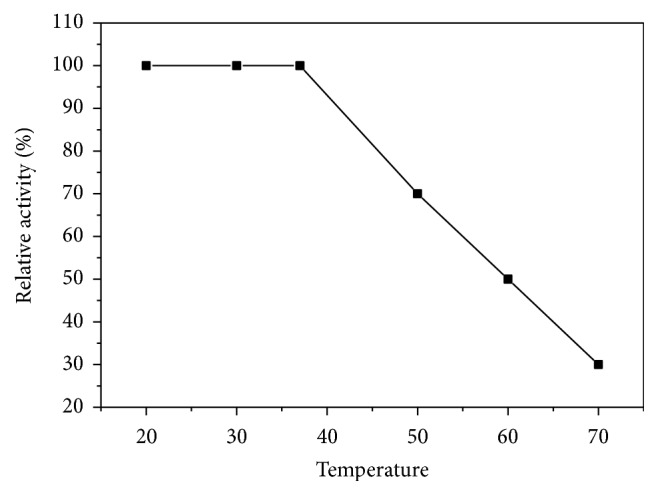
Thermal stability of asparaginase I from* P. vulgaris*. The reaction mixture was preincubated at various temperatures for 60 min prior to substrate addition, followed by cooling in an ice bath. The enzyme activity was measured using the standard assay method as previously described. Activity at zero time was taken as 100% activity.

Asparaginase I was found to have temperature optimum at 37°C (Figure 7). Similar temperature optimum of asparaginase from* V. unguiculata* (40°C) was reported [20]. This temperature was also similar to that reported for* Pseudomonas aeruginosa* and* Pectobacterium carotovorum* [32, 33]. The optimum activity of* Streptobacillus* sp. asparaginase was recorded at 35°C [26]. On the contrary, L-asparaginase from Chrombacteriaceae and* Proteus vulgaris* was observed at 20°C and 57°C, respectively [34, 35]. A nonlinear relation between asparaginase I and temperature stability was detected (Figure 8). The enzyme activity was stable up to 37°C after incubation for 1 h. Asparaginase from* V. unguiculata* was stable up to 40°C after incubation for 15 min [20]. Asparaginases from* P. carotovorum* and* C. annuum* retained their initial activity after incubation at 40°C and 45°C for 60 min, respectively [32, 33].

The effect of different metal ions on asparaginase I was examined (Table 3). The metal ions were used at the concentration of 10 mM. K^+^ was able to greatly enhance the activity of asparaginase I by 150%. In plant, K^+^-independent and K^+^-dependent asparaginases had been identified [11, 14, 36]. K^+^ acted also as enhancer on* P. carotovorum* asparaginase [32]. Ca^2+^ slightly enhanced the activity by 110%, but Cu^2+^slightly inhibited the activity of asparaginase I. In addition, Pb^2+^ and Hg^2+^ caused partially inhibitory effect on asparaginase I. However,* V. unguiculata* asparaginase was activated by Ni^2+^ and Co^2+^ and was inhibited by Mn^2+^, Zn^2+^, Ba^2+^, and Hg^2+^ [20]. EDTA as metal chelator agent caused partially inhibitory effect on asparaginase I. However, EDTA had no effect on* P. carotovorum* asparaginase [32].

## 4. Conclusions

The L-asparaginase I from* P. vulgaris* was purified in glutaminase-free form, which can reduce the possibility of side effects during the course of anticancer therapy. The enzyme showed good stability over a wide range of physiological conditions as pH and temperature. In the next step of our project, L-asparaginase I from* P. vulgaris* will be used as a potential candidate for treatment of acute lymphoblastic leukemia.

## Figures and Tables

**Table 1:** Purification scheme of asparaginase from *Phaseolus vulgaris* cv. Giza 6 seeds.

Step	Total protein (mg)	Total activity (units^*∗*^)	S.A. (units/mg protein)	Fold purification	Recovery %
Crude extract	24	940	39	1	100
Sucrose concentration	6	468	78	2	50
Chromatography on DEAE-Sepharose					
0.0 M KCl (asparaginase I)	1.8	192	106	2.7	20
0.1 M KCl (asparaginase II)	1.6	50	31	0.8	5.3
0.2 M KCl (asparaginase III)	1.2	24	20	0.51	2.5
Gel filtration on Sephacryl S-200					
Asparaginase I	0.15	127	846	21.7	13.5

^*∗*^One unit of L-asparaginase activity is defined as the amount of the enzyme that liberates 1 *µ*mol of ammonia/min.

**Table 2:** Relative activities of asparaginase I from *P. vulgaris* toward a number of asparagine and glutamine analogues at 20 mM concentration.

Substrate	% relative activity
L-Asparagine	100
D-Asparagine	2
DL-Asparagine	30
L-Glutamine	N.D.
L-Aspartic acid	2
L-Glutamic acid	1

N.D.: not detected.

**Table 3:** Effect of metal ions and EDTA at 10 mM concentration on asparaginase I activity.

Metal	% relative activity
Control	100
K^+^	150
Ca^+2^	110
Cu^+2^	94
Pb^+2^	77
Hg^+2^	33
EDTA	62
